# Topical Treatments for Localized Neuropathic Pain

**DOI:** 10.1007/s11916-017-0615-y

**Published:** 2017-03-07

**Authors:** Roberto Casale, Z. Symeonidou, M. Bartolo

**Affiliations:** 1 Department of High Technology Rehabilitation & Pain Rehabilitation Unit, Habilita Care and Research Hospitals, Via Bologna 1-24040, Zingonia di Ciserano (BG), Italy; 2 Department of Physical and Rehabilitation Medicine, General Hospital of Attica “KAT”, Athens, Greece; 3 Department of Rehabilitation, Neurorehabilitation Unit, Habilita, Zingonia di Ciserano (BG), Italy

**Keywords:** Localized neuropathic pain, Topical lidocaine, Topical ketamine, Topical baclofen, Topical amitriptyline, Topical clonidine

## Abstract

**Purpose of Review:**

Topical therapeutic approaches in localized neuropathic pain (LNP) syndromes are increasingly used by both specialists and general practitioners, with a potentially promising effect on pain reduction. In this narrative review, we describe the available compounds for topical use in LNP syndromes and address their potential efficacy according to the literature.

**Recent Findings:**

Local anaesthetics (e.g., lidocaine, bupivacaine and mepivacaine), as well as general anaesthetic agents (e.g., ketamine), muscle relaxants (e.g., baclofen), capsaicin, anti-inflammatory drugs (e.g., diclofenac), salicylates, antidepressants (e.g., amitriptyline and doxepin), α2 adrenergic agents (e.g., clonidine), or even a combination of them have been tested in various applications for the treatment of LNP. Few of them have reached a sufficient level of evidence to support systematic use as treatment options.

**Summary:**

Relatively few systemic side effects or drug–drug interactions and satisfactory efficacy seem to be the benefits of topical treatments. More well-organized and tailored studies are necessary for the further conceptualization of topical treatments for LNP.

## Introduction

The International Association for the Study of Pain (IASP) established a clear taxonomy of nociceptive and neuropathic (NP) pain as two different types of pain in 1994 [1]. The distinction between them is based on causality. The former originates from nociceptive receptor activation, while the later is directly linked to a lesion or disease of either the central or the peripheral nervous systems [2]. NP features also vary according to the location of the lesion within the nervous system, with the general rule that a more peripheral lesion has more localized symptoms and signs.

The term localized neuropathic pain (LNP) has been proposed to describe a type of NP that is ‘characterized by consistent and circumscribed area(s) of maximum pain, associated with negative or positive sensory signs and/or spontaneous symptoms characteristic of neuropathic pain’ [3], and it is felt in superficial tissues [4•].

Within the definition of LNP, there is a rationale for use of topical agents in this type of NP. Indeed, some topical treatments have already been mentioned in several guidelines for the pharmacological treatment of NP, as well as in clinical reports, along with more traditional systemic treatments [5••, 6–10] as a stand-alone treatment or as an add-on therapy [11•]. Currently, the treatment of NP can include either systemic or localized therapies, with localized treatments associated with relatively fewer systemic side effects and drug–drug interactions and satisfactory efficacy [12–14].

Localized treatments are based on the pharmacological characteristics and pharmacodynamic profile of each agent [15•]. Topical applications are administered through the skin and produce a clinically useful concentration at only the site of application without systemic concentrations [9, 14, 16], while transdermal routes produce their analgesic effect by entering the systemic circulation and include the same adverse effects as systemic administration [17]. In this narrative review, we focus only on topically acting treatments, whereas treatments that are locally applied but act systemically are not included.

A variety of agents are used in the topical treatments of LNP, such as anaesthetic agents, muscle relaxants, anti-inflammatory, antidepressant drugs or even a combination of them in various applications, and they offer promising results; however, few of them have received a sufficient level of support for systematic use as treatment options [18].

In this report, we describe each topical agent separately and address the efficacy of their use, according to their individual action mechanisms, pathology and quality of studies. We defined the papers as having high (grade 1), medium (grades 2 and 3) or low (grades 4 and 5) levels of evidence according to the ‘Centre for Evidence Based Medicine for Treatment’ classification [18].

### Topical Anaesthetic Agents

In the past, EMLA was the most used form of topical anaesthetic, in which lidocaine 2.5% was associated with prilocaine 2.5% in a eutectic mixture of localized anaesthetics in a cream vehicle, causing the effects and skin penetration to be variable [19]. Currently, lidocaine in the form of 5% lidocaine medicated plaster is the most widely studied local anaesthetic agent for the treatment of LNP [20].

Lidocaine is a sodium channel blocker, and its action is much less effective in cases of LNP, where Nav 1.7 and 1.8 sodium channels have abnormal and sensitized functions after a nerve lesion and are thought to be the most important inducers of pain. Nav 1.7 and 1.8 are located on small pain fibres, and their blockage consequently reduces ectopic discharges, which raises the peripheral ectopic discharge threshold and ameliorates the pain transduction [21]. Compared with EMLA, lidocaine plaster does not affect large myelinated fibres and thus does not cause any paraesthesia and/or numbness [22]. The plaster may have other peripheral actions through a desensitizing effect of TRPA1 channels, contributing to ‘non-anaesthetic analgesic’ effects [23]. Therefore, the reduction in peripheral sensitization could be attributed to both the blocking of pathological sodium channel expression and desensitization of TRPA1 channels. Finally, a passive protective action of the plaster itself has been reported in terms of reducing mechanical stimuli, which can trigger pain sensations [24, 25].

The application of 5% lidocaine medicated plaster has shown important effectiveness for the treatment of post-herpetic neuralgia (PHN) [26–29] and diabetic polyneuropathy (DPN) [24, 30], which has been supported by high levels of evidence studies. The use of 5% lidocaine medicated plaster has shown efficacy, which is not supported by high level evidence, except for cases of neuropathic orofacial pain syndrome (NOP) for which only a case report [31] and a case series [32] are available. Furthermore, medium-level evidence is available for efficacy in patients with LNP due to traumatic peripheral nerve injuries [33] and LNP in the area of cervical radiculopathy [34].

Other anaesthetic agents used for the topical treatment of LNP include bupivacaine and mepivacaine. In humans, bupivacaine has been suggested to have a prolonged duration of action, compared with lidocaine [35]. The use of mepivacaine, a structural analogue of bupivacaine, showed laboratory superiority in reducing pressure and heat hyperalgesia in rats with HIV-related NP, compared to lidocaine, which affected only heat hyperalgesia in a concentration-dependent manner [36]. However, its use for LNP remains on an experimental basis.

### General Anaesthetic Agents

Ketamine is a parenteral anaesthetic providing its analgesic effects in anaesthetic subdoses. The main use of ketamine is in general anaesthesia, producing analgesia, amnesia, unconsciousness and akinesia in a dose-dependent mode [37]. Its topical application for LNP relies on a NMDA receptor blockade in a non-competitive fashion, and pain modulation occurs via the blocking of glutamate production. Ionotropic glutamate receptors are expressed on peripheral afferent nerve terminals and the membranes of unmyelinated peripheral axons in response to local inflammation [38, 39]. Experimental data in animals suggested that NMDA and non-NMDA glutamate receptor expression is linked to hyperalgesia and allodynia [40, 41].

Only a few studies have described the results after the topical application of ketamine alone, as the agent is more commonly used in combination with other drugs [42–47]. The isolated use of 1% ketamine ointment in patients with PHN showed no significant difference between the active group and the placebo in a double-blinded RCT crossover study in patients [48]. Furthermore, according to another RCT, the topical application of 5% ketamine gel was not proven effective in cases of DPN [49]. Two more studies with a medium level of evidence suggested the inefficacy of 0.5 and 1% ketamine after topical application in patients with PHN, DPN and post-traumatic NP [50, 51]. On the contrary, good results were obtained from an open label study of patients with PHN treated with a topical application of 0.5% ketamine [52] and in a case series including patients with complex regional pain syndrome (CRPS; formerly described as reflex sympathetic dystrophy (RSD)), lumbar radiculopathy and PHN [53]. Reduced allodynia and hyperalgesia have been reported after topical ketamine 10% application in patients with CRPS in an RCT [54] and several case series and case reports using lower concentrations [55, 56].

### Muscle Relaxants

Baclofen is a muscle relaxant traditionally used for the systemic treatment of spasticity. The topical application of baclofen in cases of LNP offers the potential of pain reduction due to its GABAB receptor agonist features. GABAB receptor activation entails slow and prolonged inhibitory transmission, mediated by changes in membrane permeability due to an intracellular increase in K^+^ and a decrease in Ca^2+^ ions [57]. Except for the central nervous system, GABAB receptors are found in cutaneous layers on C fibres and keratinocytes, and these receptors thus provide a new target for the topical treatment of LNP [58].

Until now, baclofen is more commonly used in combination with other topical agents [44, 46, 47, 59, 60]. Effectiveness of single subcutaneous Baclofen 0.01% in reducing thermal hyperalgesia was investigated in mice with advanced skin cancer and mixed nociceptive and neuropathic pain [61]. Since then, in only two studies involving humans, baclofen 5% was used as a single topical agent for the treatment of NP in a case with NP-related to acromegaly as monotherapy [62•] and as an add-on therapy in a case with lumbar hernia-derived NP [63•]. In both studies, topical baclofen application was shown to be effective in pain relief. Studies with a higher level of evidence are not available.

### Capsaicin and Analogues

Capsaicin is a natural vanilloid derived from the capsicum plant. It selectively binds to the transient potential vanilloid receptor 1 (TRPV1), a well-characterized Ca^2+^ permeant polymodal receptor expressed on Aδ and C-nerve fibres and on the central nervous system. It releases substance P and causes transient depolarization via sodium and calcium influx. Chronic exposure to capsaicin overstimulates and desensitizes its receptors causing defunctionalization [64, 65]. The persistent analgesic effect of topical capsaicin can also be explained by causing reversible nerve degeneration, including autonomic fibres [66, 67], as well as the depletion of substance P at nervous afferent endings [64].

Regarding its efficacy, a low concentration capsaicin patch of 0.025 or 0.075% does not seem to be particularly effective in the treatment of different forms of chronic neuropathic pain based on a high level of evidence [68]. On the contrary, a subsequent high level of evidence systematic review of six studies and a total of 2000 patients reported that the topical use of an 8% capsaicin patch was more effective than a ‘placebo’ by rendering 30–50% significant post-treatment pain relief in patients with PHN and HIV-distal sensory polyneuropathy (HIV-DSP), an efficacy that was not achieved with a low-dose capsaicin patch [69]. A high-dose capsaicin application also proved useful for cervical and lumbar-related NP in a medium level of evidence study [70]. An open label, multicentre clinical trial, aiming to compare the efficacy of high-concentration capsaicin with conservative oral pregabalin therapy in peripheral NP, concluded that the capsaicin 8% patch achieved non-inferior pain relief compared to an optimized dose of pregabalin with a more rapid onset of action, fewer systemic adverse responses and better treatment satisfaction in patients with PHN, post-traumatic nerve injury and non-diabetic painful peripheral neuropathy [71]. Regarding DPN, a medium level of evidence prospective observational study of 91 cases treated with a single high-dose capsaicin patch showed a significant reduction in pain that persisted by week 12 in 34% of the patients [72]. A stronger level of evidence was suggested several years ago [73]; however, its broader local application in DPN was not suggested because the nerve fibre degeneration was caused by capsaicin [67].

Among all topical agents used for the treatment of NP, a high-dose 8% capsaicin patch was more likely to produce topical side effects, which included mild to moderate transient burning in the area of application, pain, erythema, pruritus, papules, swelling and dryness [74]. Recent data introduced a new potential superior topical analgesic agent, namely olvanil. Olvanil is a synthetic non-pungent analogue of capsaicin, and laboratory data indicated a higher analgesic effect via directly desensitizing TRPV1 channels, with fewer side effects in topical applications [75].

### Non-steroidal Anti-inflammatory Drugs

Peripheral nerve injury induces an inflammatory response with increased prostaglandin production, which enhances sodium currents and calcium influx in peripheral nociceptive neurons and increases central neurotransmitter release and the depolarization of second-order nociceptive neurons [76]. The topical application of non-steroidal anti-inflammatory drugs (NSAIDs) currently plays a crucial role, as they intervene with the inflammatory pathway and interrupt its progression [77]. When NSAIDs are applied topically, a high concentration is observed in the dermis and muscles (equivalent to that obtained via the oral route). The minimal systemic absorption of the active substance should not be totally excluded, although plasma concentrations were lower than 5% of those achieved by systemic delivery. Topical application proved to be completely safe, without topical side effects [78].

Diclofenac has great cyclooxygenase-2 (COX-2) inhibition, whereas an additional effect on peripheral nociceptors has been reported [78] along with the action of TRPV1 and TRPA1 receptors [79]. Its efficacy has been described experimentally for the treatment of neuropathic orofacial pain, with good results after 5% topical diclofenac administration [80]. Recently, a medium level of evidence study also introduced satisfying results in pain relief for patients with PHN and CRPS after the topical application of 1.5% diclofenac [81]. Ibuprofen and ketoprofen have also been administered topically in cases of NP syndromes, though always in combination with other agents [60, 82]. To the best of our knowledge, no other NSAID drug, except for diclofenac, has been used topically as a single agent for the treatment of LNP.

Salicylates also interfere with the inflammatory pathway, presenting strong analgesic features. The topical application of a mixture of aspirin and diethylether has been tested in patients with acute herpetic neuralgia and PHN, achieving excellent results of pain relief, healing acceleration and prevention of the development of PHN after the acute infection stage. The above findings were confirmed twice, with two medium level of evidence studies [83•, 84•]. In both studies, the topical analgesic effect of the mixture of aspirin and diethylether proved to be superior to the placebo group and converse to the topical application of diclofenac or indomethacin. Furthermore, topical application is linked to better pain relief results in comparison with the oral route [85, 86].

### Antidepressant Agents

Amitriptyline is a tricyclic antidepressant agent, which acts on many sites, either in the central nervous system by inhibiting neuronal reuptake of norepinephrine and serotonin or in the periphery by the blockade of Na^+^, K^+^, Ca^2+^ voltage-gated ion channels 15–17 and muscarinic, cholinergic, nicotinic, histaminergic, α2-adrenergic, adenosine and NMDA receptors [87–92]. The topical application of amitriptyline in several concentrations for the treatment of a variety of LNP syndromes has been tested, with ambiguous results, regarding not only the efficacy but also the site of action. Studies of single topical applications of 1–5% amitriptyline in various aetiologies of NP, including PHN, DPN, post-traumatic NP and painful peripheral neuropathy, did not show statistical significance in the reduction of pain with a medium level of evidence [50, 51, 93]. On the contrary, other case reports describe some efficacy of topical 5–10% amitriptyline applications in DPN, chronic idiopathic axonal polyneuropathy (CIAP), CRPS and post-traumatic neuropathic pain with a low level of evidence [94–96]. Data from these studies indicated that a high concentration of 10% amitriptyline might be more effective in pain relief; however, it seems to be related to systemic side effects [95]. Until now, the latest systematic review did not support the use of topical amitriptyline for the treatment of LNP, due to a lack of robust evidence [97].

Doxepin is another tricyclic antidepressant agent applied topically in cases of NP. Its efficacy in pain relief is demonstrated by medium and low levels of evidence for chronic NP and CRPS, respectively [98, 99], but no recent studies are available in the literature.

### α2 Adrenergic Agents

Clonidine is an α2-adrenergic receptor agonist that is generally approved for the treatment of hypertension. Target receptors for clonidine are the α2 receptors, located centrally in the brain and spinal cord and peripherally in the dorsal root ganglia, on sensory neurons [100, 101] and on nociceptors [102]. The activation of these G-protein coupled receptors downregulates adenylate cyclase and other second messengers related to the initiation and maintenance of the abnormal excitability of nociceptors [103]. Clonidine is also an imidazoline-receptor agonist, located peripherally on peripheral nerve endings. The activation of I2-imidazoline receptors may be responsible for additional mechanisms of the analgesic activity of topical clonidine applications [104]. The efficacy of 0.1% topical clonidine application has been evaluated in cases of DPN, providing a possible contribution to pain relief with no systemic absorption, with a medium level of evidence [105–107].

### Other Agents

Ambroxol is a well-tolerated agent commonly used for the treatment of respiratory disorders via its secretolytic, secretomotor and local anaesthetic effects in cases of pharyngalgia [108]. Regarding mechanisms of action, ambroxol is a potent voltage-dependent sodium channel blocker, blocking these channels approximately 40 times more strongly than lidocaine [109]. Moreover, it seems to be a preferential blocker of sodium channel subtype Nav 1.8, which is mostly expressed in nociceptive C-fibre neurons [110–112]. The drug’s efficacy has been tested experimentally in conditions of chronic pain, with good results in pain relief [113–115]. Recently, a stand-alone case series showed promising results, with significant effectiveness of topical applications in seven patients with LNP syndromes [116•]. The authors also stated a potential contribution of the agent in diminishing allodynia, strongly suggesting further research for the systematic use of ambroxol in NP treatment [116•].

Features of the abovementioned topical agents used for the treatment of LNP are summarized in Table 1.

**Table 1:** Topical agents for the treatment of LNP

Topical agent (active compound content)	Mechanism of action	Site of action	Pathology	Efficacy, level of evidence (references)
Lidocaine 5%	- Anaesthetic features: Nav channels blockade (especially 1.7 and 1.8) - Non-anaesthetic features: TRPA1 blockade	Aδ and C fibres	PHN; DPN Post-traumatic NP; cervical RP NOP	E, HLE [24, 26–30] E, MLE [33, 34] E, LLE [31, 32]
Ketamine: - 0.5–5% - 0.5% - 0.093–9.33 mg/kg - 10% - 10–50 mg/ml; 0.25–1.5%	NMDA blockade with consequent reduction of glutamate production	Aδ and C fibres	PHN; DPN; post-traumatic NP PHN CRPS, PHN and RP CRPS CRPS	NE, MLE [48–51] E, MLE [52] E, LLE [53] E, MLE [54] E, LLE [55, 56]
Baclofen 5%	GABAb receptor agonist	C fibres	NP related to: acromegaly; RP	E, LLE [62•, 63•]
Capsaicin 8%	TRPV1 receptor agonist with reversible superficial degeneration	Aδ and C fibres	PHN; HIV-DSP Cervical RP; lumbar RP DPN	E, HLE [69] E, MLE [70] E, H&MLE [72, 73]
Diclofenac 1.5%	- Topical inflammation - Topical antinociception: TRPV1, TRPA1	- Superficial tissues - Aδ and C fibres	PHN; CRPS	E, MLE [81]
Salicylates	Topical inflammation	Superficial tissues	AHN and PHN	E, MLE [83•, 84•]
Antidepressants: • Amitriptyline - 1–5% - 5–10% • Doxepin 3.3%	Blockade of Na^+^, K^+^, Ca^2+^ channels, muscarinic, cholinergic, nicotinic, H^+^, α2, adenosine, NMDA receptors	Aδ and C fibres	PHN; DPN; post-traumatic NP; peripheral neuropathy CIAP; CRPS; post-traumatic NP Chronic NP; CRPS	NE, MLE [50, 51, 93] E, LLE [94–96] E, M&LLE [98, 99]
Clonidine 0.1%	- α2 receptor agonist - I2 imidazoline receptor agonist	Aδ and C fibres	DPN	E, MLE [105–107]
Ambroxol 20%	Nav channels blockade (especially 1.8)	C fibres	LNP	E, LLE [116•]

*E* effective, *NE* non-effective, *HLE* high level of evidence, *MLE* medium level of evidence, *LLE* low level of evidence, *PHN* post-herpetic neuralgia, *DPN* diabetic peripheral neuropathy, *NP* neuropathic pain, *RP* radiculopathy, *NOP* neuropathic orofacial pain, *CRPS* complex regional pain syndrome, *HIV*-*DSP* HIV distal sensory polyneuropathy, *AHN* acute herpetic neuralgia, *CIAP* chronic idiopathic axonal polyneuropathy, *LNP* localized neuropathic pain

## Discussion

Localized neuropathic pain is a recently described form of peripheral neuropathic pain [3, 4•]. Its description and the definition of the parameters in which this term can be applied gave a stronger rational basis for topical treatments, especially in the cases of elderly people with low compliance to the oral consumption of drugs and in patients under polypharmacy. Topical treatments can be applied as add-on therapy without overcharging the patient with other orally administered drugs [11•] and have been associated with relatively fewer systemic side effects and drug–drug interactions, satisfactory efficacy and better compliance [12–14].

According to current guidelines for the treatment of peripheral NP, only two of the topical agents of this narrative review are included in the formal treatment of NP, namely, the lidocaine medicated plaster and the capsaicin patch. Previous recommendations suggested the use of lidocaine medicated plaster as the first-line treatment for postherpetic neuralgia, especially in the elderly, and the capsaicin patch as the second- or third-line treatment for postherpetic neuralgia [8, 9] or the application of the capsaicin patch as the second-line treatment for all types of peripheral neuropathic pain except for trigeminal neuralgia [7]. However, in a recent update of NeuPSIG guidelines, lidocaine patches were not recommended as the first-line treatment because of the weak final quality of evidence. Although, due to an excellent safety profile, high values and preferences, they were proposed with a weak grade of recommendation for general use as the second-line of treatment for peripheral NP [5••]. The same update proposed capsaicin patches as the second-line of treatment for peripheral NP because of the high quality of evidence, but modest effect size, training requirements and potential safety concerns on sensation with long-term use [5••].

The application of topical agents has shown good results in LNP; however, the current lack of a high level of evidence in the related studies prevents a more systematic use. Many of the studies cited in our review strongly support a better and more widespread use of various topical compounds. Diversity in study designs, concentrations and combinations of the substances, pathologies and duration of treatments and follow-up of each study creates a heterogeneous sample of available information. All these variables do not show any robust treatment indications, leading to a lack of guidelines on topical treatments.

The need of tailored protocols in large RCTs with a congruous number of patients, proper randomization, large follow-up duration and other indicators as the number needed to treat (NNT), seems to be of high importance for the further conceptualization of the topical treatment of NP. Current data suggested that the elderly population and polymedicated patients with LNP are two population subgroups that could have the most relevant benefit from the use of topical treatments.

We strongly suggest that the separate evaluation of the efficacy of each agent should precede the evaluation of combinations of agents. Until now, mixtures of topical agents have been used based either on previous knowledge about the potential efficacy and mechanisms of action or more arbitrarily. The identification of the effect of each topical compound separately can avoid several misleading circumstances regarding cost- and time-wasting trials including three to six different compounds, with possibly uncertain levels of results.

## Conclusion

In conclusion, the field of topical treatments for LNP offers a challenging area of research, with a possibly high level of potential efficacy. As the current trend indicates ongoing interest in investigational and experimental agents with plausible peripheral mechanisms of action, more well-organized and tailored studies are necessary to broaden our treatment options.
